# Supplementary material for: PredNTS: Improved and Robust Prediction of Nitrotyrosine Sites by Integrating Multiple Sequence Features
Source: Int J Mol Sci. 2021 Mar 8;22(5):2704. doi: 10.3390/ijms22052704 (PMC7962192; doi:10.3390/ijms22052704)
Supplement: Supplementary file 1 [file ijms-22-02704-s001.pdf]

Supplementary Information

PredNTS: Improved and Robust Prediction of Nitrotyrosine Sites by Integrating Multiple Sequence Features

Andi Nur Nilamyani, Firda Nurul Auliah, Mohammad Ali Moni, Watshara Shoombuatong, Md Mehedi Hasan, and Hiroyuki Kurata

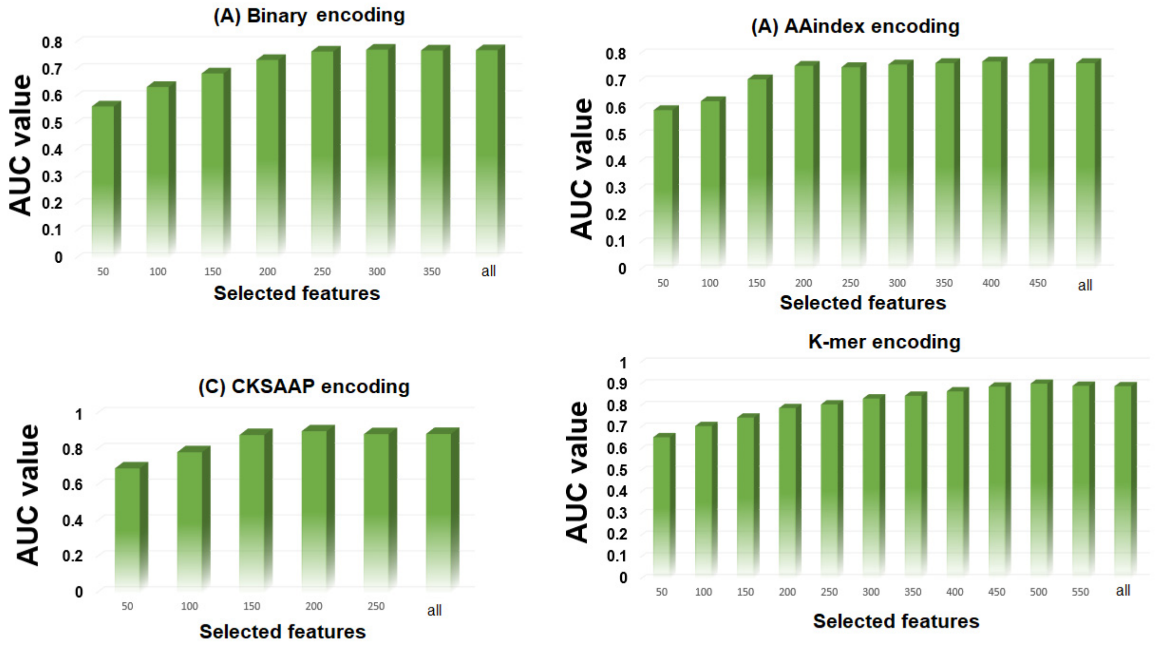

Figure S1. AUC value with respect to selected features by RFE for the four encoding schemes.
